# Spinal Subdural Empyema Successfully Treated With Conservative Therapy: A Case Report

**DOI:** 10.7759/cureus.101304

**Published:** 2026-01-11

**Authors:** Hiroki Wakiya, Daigo Arimura, Naomu Sawada, Chikara Ushiku, Mitsuru Saito

**Affiliations:** 1 Department of Orthopedic Surgery, The Jikei University School of Medicine, Tokyo, JPN

**Keywords:** conservative management, medical imaging, spinal epidural abscess, spinal infection, spinal subdural empyema

## Abstract

A 51-year-old woman presented to our hospital with septic shock. Magnetic resonance imaging (MRI) revealed a spinal epidural abscess and a spinal subdural empyema (SSE). Surgery in the prone position was considered impossible because of the patient’s recent history of cardiac surgery. Therefore, the patient was treated conservatively with antibiotics. Treatment was successful, and the patient was discharged without any neurological complications. Because of its high risk of mortality, subdural empyema is almost always treated by surgery. However, we have encountered a case in which conservative management alone was successful. Magnetic resonance images revealed two areas of dural tear. Patients with multiple tears may be candidates for conservative therapy.

## Introduction

Spinal subdural empyema (SSE), first described by Sittig in 1927, is a rare pathology, and its exact incidence is unknown [1,2]. It is a loculated collection of purulence between the dura mater and the arachnoid mater [1,3]. The lack of barriers in the spinal subdural space increases the risk of the infection spreading, leading to a rapid and extensive colonization [1]. Delayed treatment usually results in the deterioration of neurological function with potentially lethal consequences [4]. Therefore, prompt diagnosis and treatment are crucial. Early surgical drainage, followed by antibiotic therapy, is generally recommended [3,5], and there have been reports of permanent neurological deficit/death after conservative nonoperative management [3,5,6]. However, in the case of SSE described here, surgery was expected to be difficult due to a recent thoracotomy. We report a rare case of spinal subdural empyema successfully treated with antibiotic therapy alone, without surgical drainage and without any neurological sequelae.

## Case presentation

The patient was a 51-year-old woman with a history of lumbar spinal stenosis who had received lumbar nerve root block injections on four occasions at a local clinic in the previous year. She presented with a fever of 40°C that had persisted for six days before admission. She had initially visited a local hospital, where she was diagnosed with tonsillitis and prescribed antibiotics. However, the fever persisted, and she developed altered consciousness, prompting her transfer to our emergency department for further evaluation and management.

**Figure 1:**
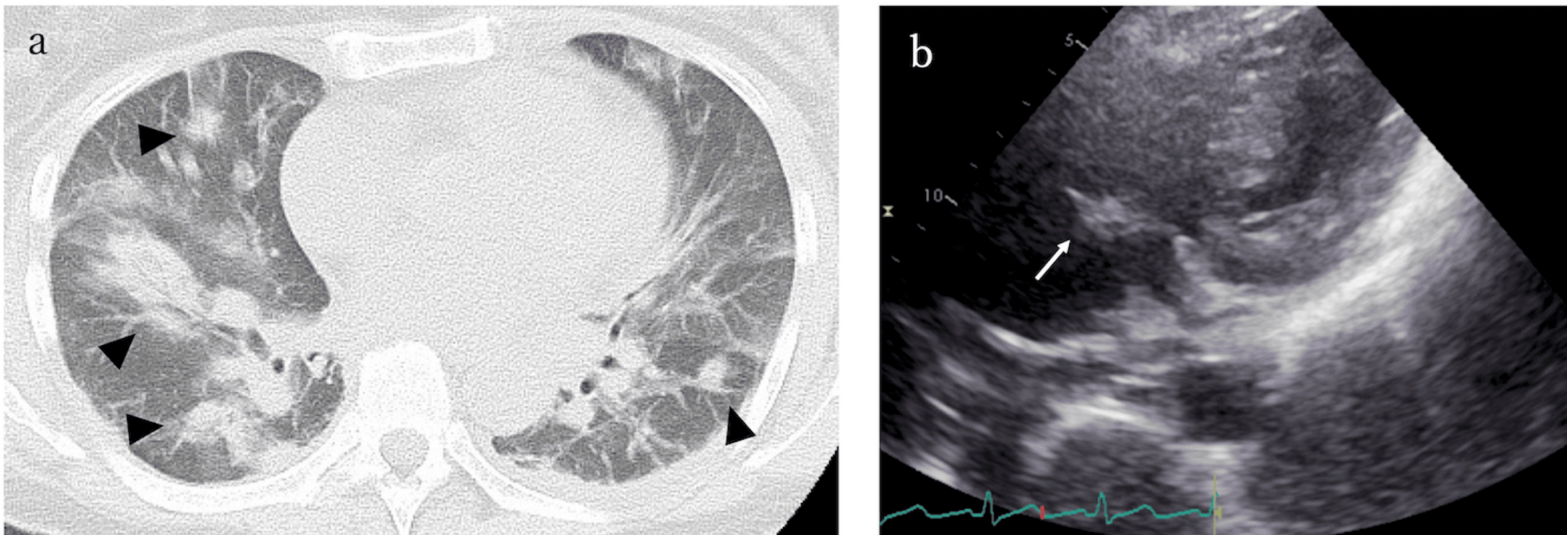
Imaging findings (a) Plain chest computed tomography showing multiple nodular shadows in the lungs. (b) Echocardiogram showing a vegetation attached to the tricuspid valve

On admission, the patient had mildly altered consciousness, fever, and increased oxygen demand. Neurological examination revealed no sensory disturbance, muscle weakness in the lower limbs, bladder or rectal dysfunction, or abnormal tendon reflexes. Laboratory tests revealed elevated inflammatory markers (white blood cell count, 20.1 × 10^3^/μL; C-reactive protein, 28.28 mg/dL). Blood and urine cultures were positive for *Staphylococcus aureus*. Chest computed tomography (CT) revealed multiple nodular opacities consistent with pneumonia (Figure 1a). She was admitted to the internal medicine department and started on antibiotic therapy. Antibiotic therapy was initially started with broad-spectrum meropenem at a dose of 1 g every 12 hours in combination with vancomycin and was de-escalated to cefazolin at 2 g every eight hours, adjusted according to renal function and antimicrobial susceptibility results. On day 8, echocardiography revealed a vegetation attached to the tricuspid valve (Figure 1b).

**Figure 2:**
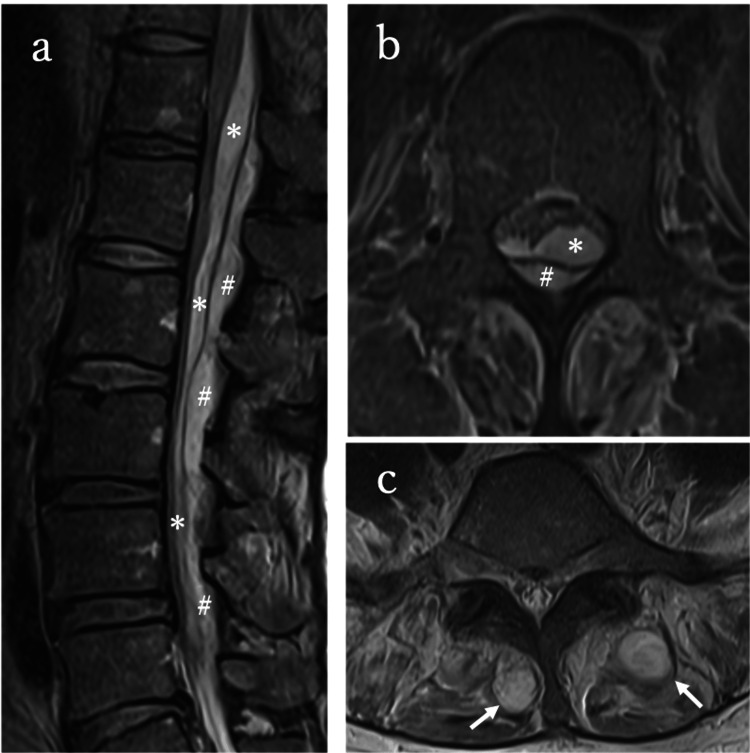
Pre-treatment MRI (a) Sagittal T2-weighted image showing subdural (*) and epidural (#) empyema. (b) Axial T2-weighted image showing subdural and epidural empyema. (c) Axial T2-weighted imaging showing a multifidus muscle abscess MRI: magnetic resonance imaging

Given the size of the vegetation, cardiac surgery was performed on day 12, and a pacemaker was implanted. Magnetic resonance imaging (MRI) of the spine performed on day 11 to investigate lower back pain revealed subdural and epidural empyema at T12-L4 and a multifidus muscle abscess (Figure 2).

**Figure 3:**
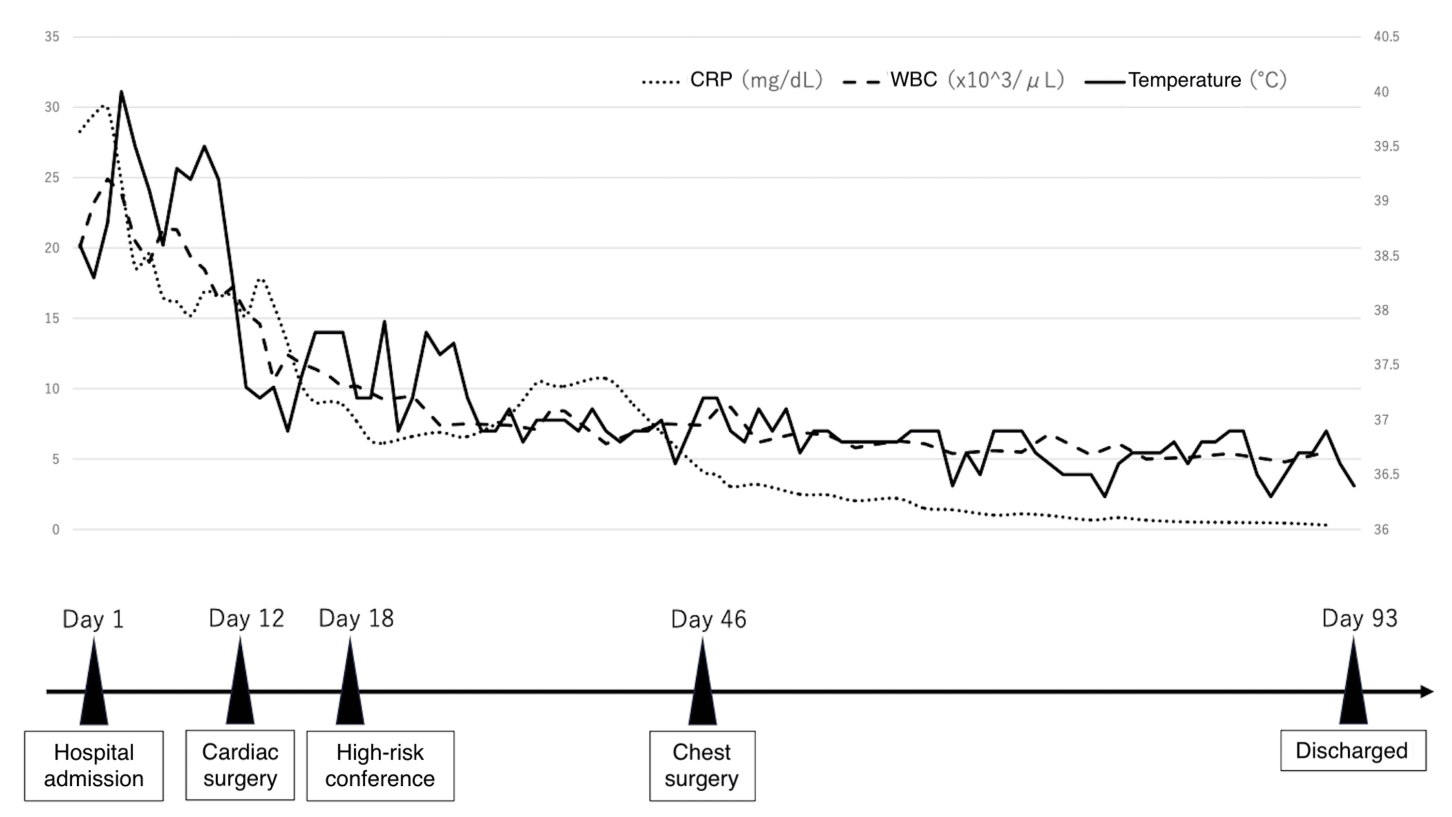
Time course of treatment The patient’s subdural empyema could not be treated surgically because of previous cardiac surgery. Blood test results improved on antibiotic therapy with no abnormal neurological findings. The patient was discharged home after a hospital stay of 93 days CRP, C-reactive protein; WBC, white blood cell

A multidisciplinary high-risk conference was convened on day 18 to discuss the need for surgical intervention to treat the spinal infection in this case. Surgical intervention was deferred, and conservative management was continued, as prone positioning in the immediate postoperative period following thoracotomy was judged to pose a high risk of pressure and mechanical stress on the surgical site. Other reasons were the favorable response to antibiotic therapy since admission and the absence of neurological deficits. Emergent surgical intervention, such as a laminectomy and a dural incision, was planned should any neurological deterioration occur during the treatment course. Conservative management with antibiotics led to clinical improvement. On hospital day 46, the patient underwent thoracoscopic debridement for empyema. The antibiotics were transitioned to oral administration by week 9. A total of 12 weeks of antibiotic therapy was completed, with no recurrence of elevated inflammatory markers. No neurological impairment was observed during treatment. She was discharged on day 93 in a stable condition (Figure 3).

During outpatient follow-up, blood tests did not reveal any signs of recurrence of infection, and no neurological symptoms were observed. At the one-year follow-up, the patient remained asymptomatic and showed no evidence of disease recurrence.

## Discussion

Spinal epidural empyema is considered relatively rare, although more than 1,000 cases have been reported. On the other hand, SSE, which occurs in the space between the dura mater and the arachnoid membrane, is much rarer, with fewer than 80 cases reported in adults [4,7-9].

SSE has been reported to progress through three stages, often in chronologic order but at a rate that is unpredictable [4,10,11]. Early symptoms include fever and back and neck pain, with radicular pain referred from the affected nerve roots. Patients then develop motor and sensory deficits and urinary sphincter dysfunction. In the most advanced stage, the symptoms include paralysis, incontinence, and complete sensory loss. Prompt diagnosis is important in the presence of neurological symptoms, which can have a rapid onset and be irreversible [12].

Laboratory tests are not particularly sensitive for SSE, with leukocytosis detected on presentation in only 57% of cases [10]. However, other inflammatory markers, such as the erythrocyte sedimentation rate and C-reactive protein level, have been reported to be elevated in 94.1% and 100% of cases, respectively [6]. Our patient presented with leukocytosis and high inflammatory marker levels, which may not have been caused by SSE alone, considering that the patient also had pneumonia and bacteremia. MRI with gadolinium contrast is considered the gold standard for the diagnosis of SSE [12]. Epidural empyema and subdural empyema can sometimes be differentiated on the basis of MRI alone by the presence of epidural fat, which is a typical finding in SSE [13,14].

The most common causative organism is *S. aureus*, which was also seen in this case [3]. The risk factors for SSE are similar to those for epidural empyema and include immunocompromising conditions (e.g., diabetes, chronic alcohol abuse, chronic corticosteroid use, malignancy, and human immunodeficiency virus infection), as well as active drug abuse and a recent history of a spinal procedure [4,12,15]. The pathophysiology of SSE is still unclear, but three modes of spread have been proposed, namely, hematogenous, contiguous, and iatrogenic [9,11,15-17]. Hematogenous spread is the most common mode of infection, occurring in 43% of cases, and has been reported to be caused by sepsis, pneumonia, and intravenous drug abuse [12]. Iatrogenic causes include lumbar puncture, epidural anesthesia, discography, and a dural tear during spinal surgery and have been identified in 25% of cases [9,18]. In this case, considering that a considerable period had elapsed since the lumbar nerve root block and that the SSE appeared to be at an early stage relative to the systemic infection, we speculate that the infection most likely spread via the hematogenous route. However, it remains difficult to definitively determine the route of infection.

The subdural space runs from the foramen magnum through the spine, interrupted only by penetrating vessels and nerves, and its contiguous nature allows the rapid longitudinal spread of infection [7]. Urgent surgical drainage, followed by antibiotics, is strongly recommended for subdural empyema because of its unpredictable course and the high risk of neurological sequelae [3,4,17,19].

The conservative treatment of subdural abscess has been reported to have a mortality rate of 43%-100%, whereas surgery has a mortality rate of 13%-18% and a high rate of complete or marked recovery (73%) [6,12].

**Figure 4:**
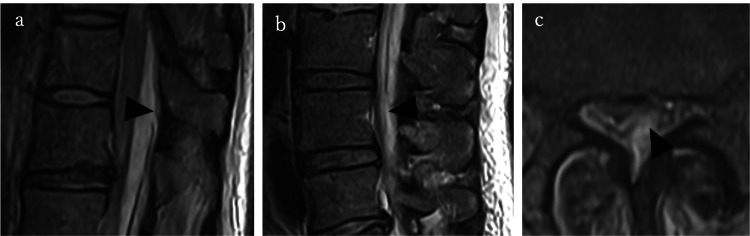
Pre-treatment MRI showing the tear in the dura mater Sagittal T2-weighted images showing (a) the cranial portion at the Th11/12 level and (b) the caudal portion at the L3/4 level. (c) Axial T2-weighted images showing the dural tear MRI: magnetic resonance imaging

No neurological impairment was observed in the present case. The patient presented with low back pain and fever only, indicating that the SSE was at an early stage. The diagnosis of SSE was relatively straightforward, with MRI clearly demonstrating fluid collections both inside and outside the dura mater, which delineated the subdural and epidural spaces (Figure 2). Although surgery was initially considered because of the severity of SSE, it was deferred in favor of continued conservative management, given the high risk associated with the patient’s recent cardiac surgery. Unlike in previous reports where conservative management was associated with poor outcomes (mortality of up to 43%), our patient recovered fully without neurological sequelae. We speculate that conservative management was successful in this case because of the spontaneous epidural drainage of the abscess. MRI demonstrated two dural tears, one on the cephalic side of the empyema and the other on the caudal side, suggesting that subdural pus may have drained into the epidural space through these defects and therefore could be treated successfully (Figure 4).

As previously described, collections of subdural fluid tend to extend longitudinally in both the cephalad and caudal directions [7]. However, the mechanism by which the collection breached the dura at two distinct sites in this case cannot be definitively identified. 

There have been a few reports of minimally invasive drainage techniques, such as CT-guided aspiration or lumbar drainage, as alternatives to open surgery in selected patients with subdural or epidural empyema [13,20]. This may have been a treatment option, considering that the prone position was not feasible. However, because the patient was on dual antiplatelet therapy following cardiac surgery, the procedure was deemed too risky.

This report has several limitations. First, a detailed postoperative imaging evaluation was not possible because MRI follow-up was precluded by pacemaker implantation. Although contrast-enhanced CT images were obtained, they were suboptimal for the assessment of the resolution of the abscess around the neural elements. Second, no direct microbiological confirmation of the subdural abscess was obtained because surgical or percutaneous drainage was not performed. Therefore, the causative organism was inferred from blood cultures rather than from the abscess itself. If material from the abscess could have been obtained for culture, it might have been possible to glean more definitive microbiological evidence for the purposes of diagnostic accuracy. Third, the mechanism by which the infection breached the dura mater at two distinct locations remains unclear. Spinal subdural empyema is generally considered to spread preferentially in the cranio-caudal direction due to the absence of anatomical barriers within the subdural space, and the dura mater itself is relatively resistant to disruption [1]. One speculative possibility is that epidural infection developed independently at two sites, each subsequently extending into the subdural space, followed by longitudinal spread and secondary connection within the subdural compartment. However, this hypothesis cannot be confirmed based on the available clinical and imaging data and remains speculative. Finally, this was a single rare case, and whether conservative management would be appropriate for other patients with SSE remains uncertain. Further accumulation of similar cases is needed to determine the appropriate selection criteria for nonoperative management. This rare case demonstrates that conservative management may achieve favorable outcomes in patients with SSE when early diagnosis, effective antibiotic therapy, and a potential route of spontaneous drainage are present.

## Conclusions

SSE is a life-threatening condition that typically requires prompt surgical drainage, with delayed intervention potentially leading to irreversible neurological deficits or death. However, this case demonstrates that conservative management alone can result in complete recovery under certain circumstances. The identification of two dural tears on MRI suggests a possible mechanism of spontaneous drainage, which may have contributed to the favorable clinical outcome.

Although surgical debridement remains the gold standard treatment for SSE, nonoperative management can be considered in carefully selected patients who have absolute contraindications to surgery and radiological evidence of communication between the subdural and epidural spaces. It is important to emphasize that even in such cases, surgery should remain the recommended treatment in principle. Conservative management should only be undertaken with multidisciplinary agreement, explicit informed consent, and close neurological monitoring, with readiness for urgent surgical intervention in the event of clinical deterioration.
